# Linking obesity with white matter microstructure highlights the importance of brainstem tracts and sex differences

**DOI:** 10.1093/braincomms/fcag026

**Published:** 2026-01-30

**Authors:** Tiril P Gurholt, Dani Beck, Irene Voldsbekk, Nadine Parker, Daniel E Askeland-Gjerde, Ann-Marie G de Lange, Dennis van der Meer, Christian K Tamnes, Paul M Thompson, Ida E Sønderby, Ivan I Maximov, Lars T Westlye, Ole A Andreassen

**Affiliations:** Section for Precision Psychiatry, Division of Mental Health and Addiction, Oslo University Hospital, 0450 Oslo, Norway; Department of Psychology, University of Oslo, 0373 Oslo, Norway; Division of Mental Health and Substance Abuse, Diakonhjemmet Hospital, 0373 Oslo, Norway; PROMENTA Research Center, Department of Psychology, University of Oslo, 0373 Oslo, Norway; Section for Precision Psychiatry, Division of Mental Health and Addiction, Oslo University Hospital, 0450 Oslo, Norway; Department of Psychology, University of Oslo, 0373 Oslo, Norway; Division of Mental Health and Substance Abuse, Diakonhjemmet Hospital, 0373 Oslo, Norway; Division of Mental Health and Addiction, Centre for Precision Psychiatry, Institute of Clinical Medicine, University of Oslo, 0450 Oslo, Norway; Section for Precision Psychiatry, Division of Mental Health and Addiction, Oslo University Hospital, 0450 Oslo, Norway; Institute of Clinical Medicine, University of Oslo, 0450 Oslo, Norway; Department of Psychology, University of Oslo, 0373 Oslo, Norway; School of Biological and Behavioural Sciences, Queen Mary University of London, E1 4NS London, UK; Department of Psychiatry, University of Oxford, OX3 7JX Oxford, UK; Division of Mental Health and Addiction, Centre for Precision Psychiatry, Institute of Clinical Medicine, University of Oslo, 0450 Oslo, Norway; School of Mental Health and Neuroscience, Faculty of Health, Medicine and Life Sciences, Maastricht University, 6200 MD Maastricht, The Netherlands; Department of Psychology, University of Oslo, 0373 Oslo, Norway; Division of Mental Health and Substance Abuse, Diakonhjemmet Hospital, 0373 Oslo, Norway; PROMENTA Research Center, Department of Psychology, University of Oslo, 0373 Oslo, Norway; Imaging Genetics Center, Mark & Mary Stevens Neuroimaging & Informatics Institute, Keck School of Medicine, University of Southern California, Marina del Rey, CA 90292, USA; Section for Precision Psychiatry, Division of Mental Health and Addiction, Oslo University Hospital, 0450 Oslo, Norway; Department of Medical Genetics, Oslo University Hospital, 0450 Oslo, Norway; K.G. Jebsen Centre for Neurodevelopmental Disorders, University of Oslo, 0450 Oslo, Norway; Department of Health and Functioning, Western Norway University of Applied Sciences, 5063 Bergen, Norway; Section for Precision Psychiatry, Division of Mental Health and Addiction, Oslo University Hospital, 0450 Oslo, Norway; Department of Psychology, University of Oslo, 0373 Oslo, Norway; Department of Medical Genetics, Oslo University Hospital, 0450 Oslo, Norway; Section for Precision Psychiatry, Division of Mental Health and Addiction, Oslo University Hospital, 0450 Oslo, Norway; Division of Mental Health and Addiction, Centre for Precision Psychiatry, Institute of Clinical Medicine, University of Oslo, 0450 Oslo, Norway; Department of Medical Genetics, Oslo University Hospital, 0450 Oslo, Norway

**Keywords:** waist-to-hip ratio, waist circumference, DTI, cardiometabolic conditions, somatic disorder

## Abstract

While obesity (body mass index ≥ 30) has been consistently associated with white matter diffusion magnetic resonance imaging (MRI) phenotypes, the contributions of common obesity phenotypes on various diffusion metrics, and the moderating effects of sex and age, require further clarification. This study aims to elucidate these body–brain connections to enhance our understanding of the comorbid link between obesity and body anthropometrics and the brain using a large-scale dataset. We analysed cross-sectional data from 40 040 participants from the UK Biobank (52.2% female; ages 44–83 years) using multiple linear regression to evaluate how obesity and body anthropometrics relate to regional white matter diffusion tensor imaging metrics (fractional anisotropy, axial diffusivity, radial diffusivity, mean diffusivity). We also examined interactions with age and sex. Our analyses revealed significant associations between individual obesity phenotypes (i.e. obesity and body anthropometrics) and diffusion tensor imaging metrics of small effects, with partial correlation coefficient |*r*| effect sizes ranging from 0.02 to 0.20 for most regions of interest with largest effects in brainstem tracts. We observed more widespread sex-by-obesity phenotypes than age-by-obesity phenotypes interaction effects on diffusion tensor imaging metrics. Our results link obesity and body anthropometrics with white matter phenotypes and suggests that shared body fat-related pathways link physical and brain health that may vary based on sex and age. Understanding these body–brain relationships, and the role of age and sex, could enhance the development and evaluation of targeted, personalized, treatment strategies for brain disorders that co-occur with obesity, although further longitudinal and intervention studies are needed to map the causal dynamics of these associations.

## Teaser

Gurholt et al. report associations between obesity and regional white matter phenotypes across commonly used diffusion magnetic resonance imaging metrics, with largest effects for brainstem tracts that interconnects the brain and body, and indications of notable age and sex variations. The observations may be important for brain and somatic disorders.

## Graphical Abstract

**Graphical Abstract:**
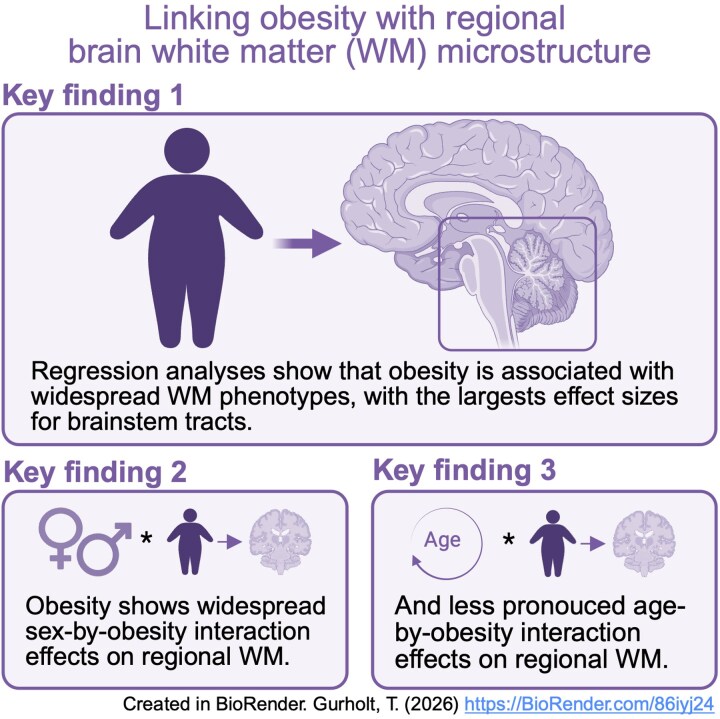

## Introduction

Obesity and its associated cardiometabolic conditions are frequently observed alongside neurological and psychiatric brain disorders.^1-3^ Research has shown that obesity (i.e. body mass index ≥ 30) and body anthropometrics^4-9^ and brain disorders^10^ are linked to brain white matter alterations, possibly due to common underlying mechanisms. There are also notable sex- and age-related variations in body fat distribution,^11^ brain white matter magnetic resonance imaging (MRI) phenotypes,^12^ and the prevalence and clinical features of common brain disorders.^3,13,14^ Therefore, gaining a deeper understanding of how commonly used indicators of excess body fat—obesity and body anthropometrics—relate to white matter phenotypes, and whether and how these relationships are influenced by age and sex, is crucial for unraveling the complex comorbidities between cardiometabolic conditions and brain disorders.

Diffusion magnetic resonance imaging (dMRI) is a widely used technique for non-invasive, *in vivo*, characterization and quantification of white matter organization and microstructure. Diffusion tensor imaging (DTI)^15^ is sensitive to the direction and magnitude of water molecule diffusion in brain tissue.^16^ From the diffusion tensor, various invariant diffusion metrics can be derived, including fractional anisotropy (FA; the degree of rotation invariant anisotropic diffusion), axial diffusivity [AD; the diffusivity along the principal eigenvector (i.e. main diffusion direction)], radial diffusivity [RD; the diffusivity perpendicular to the principal eigenvector (i.e. non-main diffusion)], and mean diffusivity (MD; the average diffusivity over eigenvectors).^16^ These metrics provide indirect and integrative insight into the underlying white matter tissue organization. Interpretation can be challenging as observed DTI variations might be due to various biological or disease processes,^17^ as well as dMRI artefacts.^16^ However, lower FA is often observed in disorders and interpreted as not favourable, while lower AD, higher RD, and higher MD may indicate axonal degeneration, demyelination, and inflammation, respectively.^16^ When analysed together, these DTI metrics provide deeper insights into tissue properties and of regional variation that may be relevant for disentangling disease processes.

Prior studies suggest that obesity and body anthropometrics [e.g. BMI, waist-to-hip ratio (WHR), and waist circumference],^4-9^ as well as other cardiometabolic risk factors,^4,18,19^ are associated with dMRI white matter phenotypes. The most consistent findings include widespread lower FA at higher BMI and WHR,^4-8^ with indications of higher FA for some brainstem tracts.^4^ A meta-analysis reported lower FA in the genu of the corpus callosum in obesity.^9^ For AD and RD, prior reports are mixed.^6^ Few larger studies exist,^4,7-9^ and they differ in focus, investigated regions, and methods. They studied obesity^9^ and body anthropometrics (BMI^4,7,8^; WHR^4,8^). All reported on FA,^4,7-9^ one reported on MD,^4^ and none reported on AD and RD. There is a need for large-scale studies that investigate obesity and body anthropometrics with regional white matter characteristics assessed using multiple DTI metrics to obtain deeper insight into the link between obesity and body anthropometrics and brain tissue properties.

Sex- and age-related differences in body shape and fat distribution^11^ and dMRI features^20-22^ might contribute to the reported links between obesity and white matter phenotypes. A review reported negative correlations between BMI and FA in several brain regions and indications of accelerated white matter ageing in obese individuals.^5^ Higher white matter brain age has been associated with higher body fat^23,24^ and other cardiometabolic risk factors,^25,26^ and one study indicated multisystem ageing with both shared and unique body and brain ageing processes.^27^ Further, interactions between body fat and sex may influence white matter microstructure,^19^ with a stronger association between higher white matter brain age and body fat in males than females,^24^ possibly reflecting that the normative range of body fat is higher for females than males.^28^ To our knowledge, no study has comprehensively investigated sex and age interactions with both obesity and body anthropometrics on regional white matter phenotypes across a range of DTI metrics.

**Figure 1:**
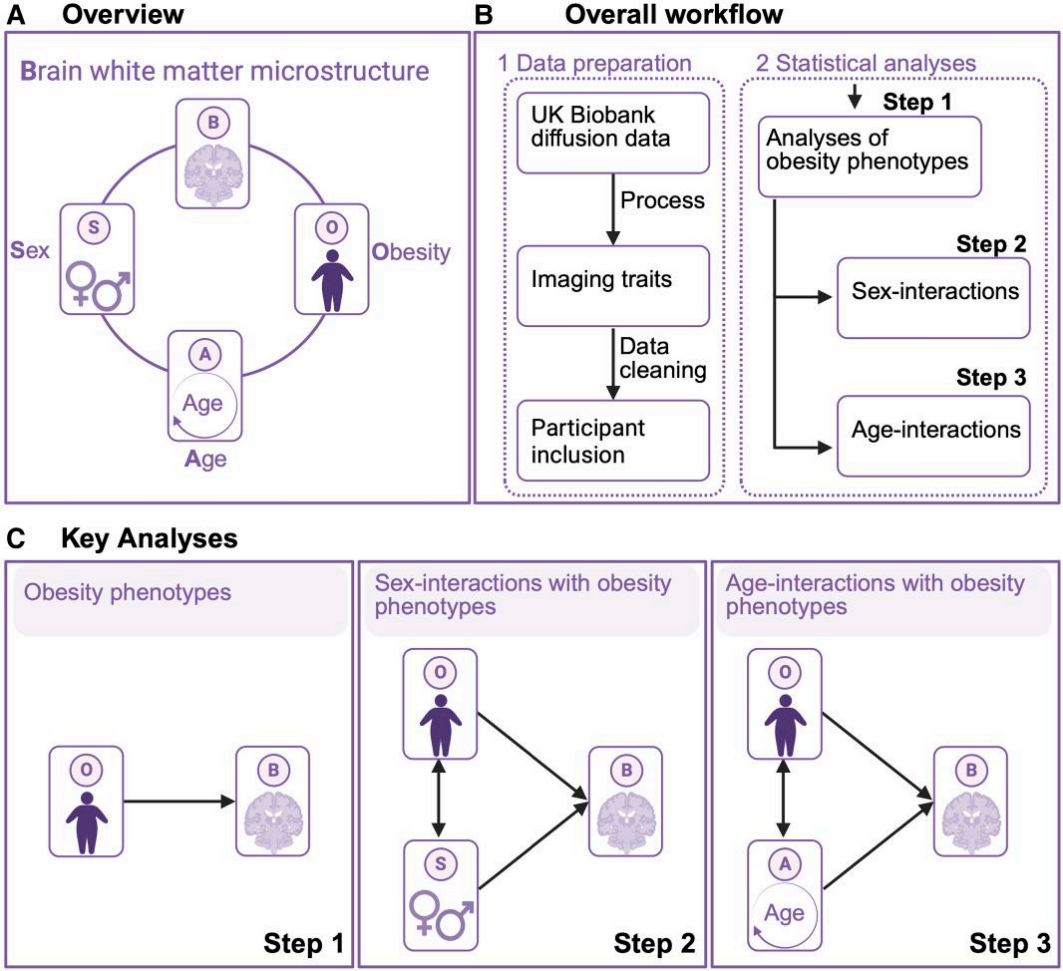
Overview of study design and analyses. (**A**) Overview of the study design targeting age, sex, and obesity phenotypes (i.e. obesity, body anthropometrics) relative to white matter microstructure. Description of the (**B**) study workflow and (**C**) key analyses (created in BioRender. Gurholt, T. (2026) https://BioRender.com/pxegtfo).

To enhance our understanding of the links between physical and brain health and of putative shared obesity-related pathways, in the largest study to date, we investigated the links between obesity phenotypes (i.e. obesity, body anthropometrics) with DTI-based brain white matter phenotypes among 40 040 middle-aged to older adults (52.2% female) from the UK Biobank (https://www.ukbiobank.ac.uk). To target putative complex interplays with sex and age, we also tested for interactions between obesity phenotypes and both sex and age on white matter phenotypes (Fig. 1). To verify the robustness of the results, we include sensitivity analyses where we adjust for self-reported cardiometabolic comorbid factors.

## Materials and methods

### Study design and participants

The UK Biobank is an epidemiological longitudinal population cohort of middle- to old-aged participants recruited from assessment sites across the UK.^29^ The baseline assessment took place during 2006–10 and included approximately 500K participants. The follow-up brain MRI assessment started in 2014 and is ongoing. We included 40 040 participants (20 904 females, 19 136 males) with brain dMRI and demographic and clinical data from the follow-up imaging assessment. We excluded participants who withdrew their informed consent (opt-out-list dated 25 April 2023).

The UK Biobank has IRB approval from the North-West Multi-center Research Ethics Committee and obtained informed consent from all participants.^29^ We obtained access to the UK Biobank resources through Application number 27412. We have approval from the Norwegian Regional Committees for Medical and Health Research Ethics (https://rekportalen.no/; Application number 2009/2485).

### Demographic and clinical data

**Table 1** summarizes the demographic and clinical data. We extracted age, sex, body anthropometrics (waist circumference, hip circumference, weight, height, BMI), and self-reported ethnicity, history of diabetes, hypercholesterolaemia, hypertension, cigarette smoking, and alcohol consumption (Supplementary Note 1). We computed WHR, and derived binary (yes/no) variables for obesity (obesity: BMI ≥ 30; non-obesity: BMI < 30) and self-reported European/non-European ethnicity (i.e. white, white British, white Irish, other white background versus other background), current cigarette smoking, and current alcohol consumption. We complemented self-reported ethnicity at MRI with data from the baseline assessment when necessary.

**Table 1:** Demographic and clinical data

	Male versus female				Participants with obesity versus non-obesity			
	Male (*n* = 19 136)	Female (*n* = 20 904)	Test	*P*-value	Obesity (*n* = 6912)	Non-obesity (*n* = 33 128)	Test	*P*-value
Women					3507 (50.7)	17 397 (52.5)	7.2	0.0074
Age (year)^a^	64.9 ± 7.8	63.7 ± 7.6	15.7	<0.0001	63.5 ± 7.6	64.4 ± 7.8	−8.9	<0.0001
Age range (year)	[4 483]	[45 83]			[44 83]	[45 83]		
European ethnicity^b^	18563 (97)	20293 (97.1)	0.2	0.6949	6697 (96.9)	32 159 (97.1)	0.6	0.4300
Cigarette smoker^b^	601 (3.1)	470 (2.2)	30.2	<0.0001	192 (2.8)	879 (2.7)	0.3	0.5877
Smoker current/previous/never	601/7049/11486	470/6432/14002			192/2626/4094	879/10855/21394		
Alcohol consumer^b^	18125 (94.7)	19 380 (92.7)	67.5	<0.0001	6394 (92.5)	31 111 (93.9)	18.8	<0.0001
Alcohol consumer current/previous/never	18 125/587/424	19 380/679/845			6394/247/271	31 111/1019/998		
Obesity^b^	3405 (17.8)	3507 (16.8)	7.2	0.0074				
Height (cm)^a^	176 ± 6.6	162.7 ± 6.2	207	<0.0001	168.6 ± 9.4	169.2 ± 9.2	−4.6	<0.0001
Weight (kg)^a^	83.3 ± 13.2	68.7 ± 12.9	112.1	<0.0001	95.3 ± 13.5	71.6 ± 11.6	135.6	<0.0001
BMI^a^	26.9 ± 3.8	25.9 ± 4.7	21.7	<0.0001	33.4 ± 3.4	24.9 ± 2.7	197	<0.0001
Waist circ. (cm)^a,c^	94.1 ± 10.5	82.6 ± 11.7	102.7	<0.0001	103.9 ± 10.4	84.8 ± 10.3	140.1	<0.0001
Hip circ. (cm)^a^	100.5 ± 7.3	100.6 ± 9.8	−1.8	0.0773	112.4 ± 8.6	98.1 ± 6.3	131.4	<0.0001
WHR^a^	0.9 ± 0.1	0.8 ± 0.1	172.6	<0.0001	0.93 ± 0.09	0.86 ± 0.08	52.7	<0.0001
Diabetic^b^	469 (2.5)	253 (1.2)	86.1	<0.0001	291 (4.2)	431 (1.3)	271.7	<0.0001
High cholesterol^b^	3195 (16.7)	2008 (9.6)	443.6	<0.0001	1209 (17.5)	3994 (12.1)	148.9	<0.0001
Hypertension^b^	4768 (24.9)	3500 (16.7)	406.8	<0.0001	2371 (34.3)	5897 (17.8)	949.4	<0.0001

*Notes:* The table reports mean ± standard deviation for continuous variables, and *n* (%) for categorical variables. Footnotes describe the applied test. Obesity is defined as BMI ≥ 30, and self-reported data was used to determine ethnicity, diagnosis of diabetes, high cholesterol, and hypertension, and current alcohol consumption and cigarette smoking.

*Abbreviations:* BMI—body mass index; circ.—circumference; WHR—waist-to-hip ratio.

^a^Welch Two Sample *t*-test.

^b^Pearson's Chi-squared test with Yates’ continuity correction.

^c^Two sample *t*-test (participants with obesity versus non-obesity only).

### MRI acquisition and post-processing

MRI acquisition details are described elsewhere.^29,30^ Briefly, 3D multimodal MRI of the brain was performed at four separate sites (Cheadle, Reading, Newcastle, or Bristol) in the UK, with similar 3T Siemens Skyra scanners, Siemens 32-channel head coils, and scanning protocols across sites.

We post-processed the available dMRI data using an optimized pipeline.^31^ We used state-of-the-art dMRI processing^32^ and corrected for noise using MP-PCA,^33^ Gibbs ringing using unring,^34^ susceptibility distortion using FSL^35^ topup,^36^ eddy-current and motion-induced distortions using FSL Eddy,^37-39^ and applied isotropic 1 mm^3^ Gaussian kernel smoothing to increase the signal-to-noise ratio using FSL fslmaths. We derived DTI scalar metrics of FA, RD, AD, and MD using a cumulant expansion of the diffusion signal up to the fourth order based on diffusion kurtosis tensors^40-42^ using MATLAB scripts (https://github.com/NYU-DiffusionMRI/DESIGNER-v1) with two different *b*-values (*b*-values: 1000 and 2000 s/mm^2^), which allowed us to extract more sensitive DTI metrics in contrast to the conventional one *b*-value estimation.^41,42^

**Figure 2:**
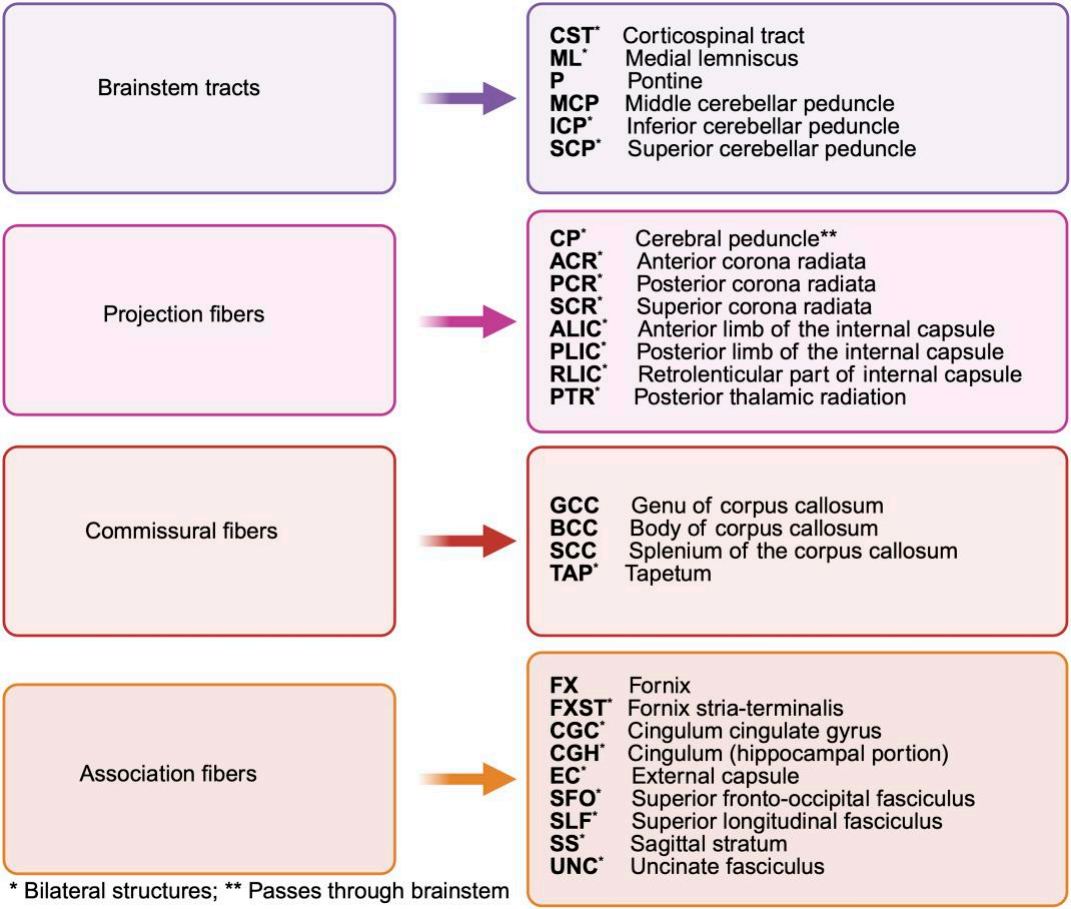
Overview of the included white matter regions of interest (created in BioRender. Gurholt, T. (2026) https://BioRender.com/a02w4fp).

We normalized DTI metrics using FSL tract-based spatial statistics (TBSS)^43^ and harmonized them using the YTTRIUM algorithm.^44^ We aligned all diffusion metrics to the *FSL FMRI58_FA template* using nonlinear transformation in *FSL FNIRT.*^31^ We derived the mean FA image of all participants, thinned it to create the mean FA skeleton, and projected the scalar diffusion maps onto the FA skeleton. In order to harmonize the obtained diffusion data, we used YTTRIUM algorithm.^44^ We extracted the 27 regions of interest (ROIs) for each diffusion map from the Johns Hopkins University (JHU) DTI atlas,^45^ and subsequently partitioned them into brainstem, projection, commissural, and association pathways (Fig. 2).

### Quality control

Based on YTTRIUM for automatic dMRI quality control,^44^ we removed 670 participants with poor dMRI data quality. We also removed five participants with incomplete diffusion data. In addition, we removed 1839 participants with incomplete demographic or clinical data, resulting in a final sample of 40 040 participants (Supplementary Fig. 1).

### Statistical analysis

We investigated the sample demographic and clinical data across and within sexes and for obesity versus non-obesity (Table 1). We compared categorical variables using the *χ*^2^-test. We evaluated continuous variables for normality by visual inspection of density plots (Supplementary Fig. 2) and compared normally distributed variables using the two-sample *t*-test/Welch approximation for equal/unequal variance. We assessed density plots of diffusion metrics for expected distribution patterns (data not shown; all reasonably normal).

In separate multiple linear regression analyses, we investigated each individual regressor of interest with the individual ROIs from the JHU atlas for the included DTI metrics (i.e. FA, AD, RD, and MD). In Step 1, we tested for associations between the individual obesity phenotypes (i.e. obesity versus non-obesity, BMI, WHR, and waist circumference) and each DTI ROI, while adjusting for covariates as follows:


$$ROI=obesity_{\;phenotype}+age+age^{2}+sex+age\times sex+age^{2}\times sex+ethnicity+site\;\forall \;ROIs,\;DTI\;metrics,\;and\;obesity\;phenotypes.$$

The symbol ∀ means *for all*. For the next two steps we used slightly modified multiple linear regression models. In Step 2, we tested for associations between obesity phenotypes-by-sex interactions and each DTI ROI, while adjusting for covariates as follows:


$$ROI=obesity_{\;phenotype}\times sex+obesity_{\;phenotype}+sex+age+age^{2}+ethnicity+site\;\forall \;ROIs,\;DTI\;metrics,\;and\;obesity\;phenotypes.$$

In Step 3, we tested for associations between obesity phenotypes-by-age interactions and each DTI ROI, while adjusting for covariates as follows:


$$ROI=obesity_{\;phenotype}\times age+obesity_{\;phenotype}+age+sex+ethnicity+site\;\forall \;ROIs,\;DTI\;metrics,\;and\;obesity\;phenotypes.$$

Lastly, we included sensitivity analyses for the analyses (Steps 1–3) where we additionally adjusted for self-reported cardiometabolic comorbid factors (i.e. diagnosis of diabetes, high cholesterol, and hypertension, and current smoking and alcohol consumption).

We implemented all statistical analyses in R (version 4.2.1; https://www.r-project.org). We included both linear and quadratic terms for age and interactions with sex, when relevant, to account for known nonlinear associations between age and white matter microstructure,^46^ and possible sex-related differences in aging trajectories. We adjusted all models for self-reported ethnicity since ethnicity is considered a relevant factor for body fat accumulation and obesity assessment.^11^ We mean-centred all continuous regressors and entered binary variables as factors. We computed the standardized partial correlation coefficient *r*-effect size directly from the *t*-statistics for continuous variables and via Cohen’s *d* for categorical variables, and the corresponding confidence intervals of the *r* effect sizes.^47^ We implemented a study-wide Bonferroni multiple comparison correction at *α* = 0.05 across *n* = 48 × 6 × 4 = 1152 independent tests, reflecting the 48 ROIs (counting bilateral ROIs twice), six main variables of interest (i.e. obesity versus non-obesity, BMI, WHR, and waist circumference, and sex- and age-interactions), and four DTI metrics, counting partly overlapping models once, yielding a study-wide significance threshold of *P* ≤ *α*/*n*  ≈ 4.3e−05. We focused on the overall patterns and the most significant findings and reported the range of *P*-values and *r-effect* sizes (absolute values are indicated by |*r*|). Some *P*-values are rounded to zero since they are approximately zero and below numeric precision (i.e. smaller value than can be represented). We report the full results in the supplemental material.

## Results

### Demographic and clinical data

**Table 1** presents the demographic and clinical data. Briefly, the sample included more females (*n* = 20 904; 52.2%; mean age 63.7 ± 7.6 years) than males (*n* = 19 136; 47.8%; mean age 64.9 ± 7.8 years), had age range 44–83 years, and consisted predominantly of self-identified white Europeans (97.0%). On average, males exhibited higher levels of anthropometric traits for all measures (except hip circumference) compared to females. Participants with obesity (*n* = 6912; 50.7% female) had, on average, lower age, higher body anthropometric traits (except lower height), fewer alcohol consumers, and higher prevalence of self-reported cardiometabolic diagnosis than non-obese participants (*n* = 33 192; 52.5% female).

### Associations between obesity phenotypes and DTI metrics

**Figure 3:**
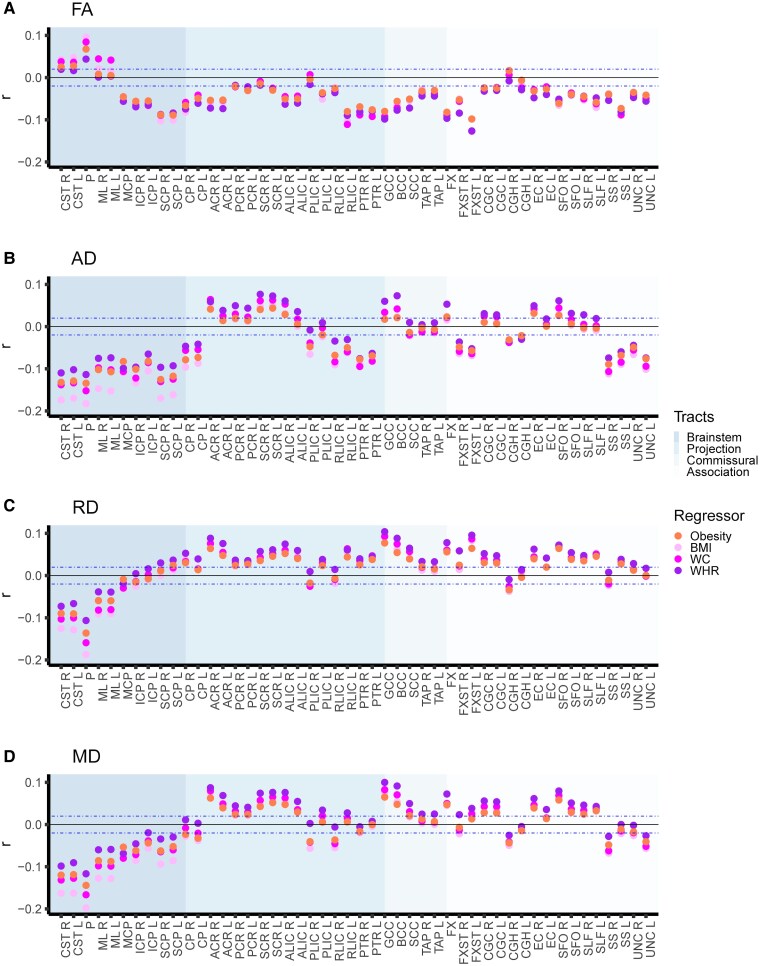
Obesity phenotypes with diffusion tensor imaging metrics. The figure shows the multiple linear regression results of (i) obesity versus non-obesity; (ii) BMI; (iii) waist circumference; and (iv) WHR separately on diffusion tensor imaging metrics for (**A**) FA, (**B**) AD, (**C**) RD, and (**D**) MD, after adjusting for age, age^2^, sex, age-by-sex, age^2^-by-sex, ethnicity, and site. We included 40 040 UK Biobank participants (*n* = 6912 with obesity; *n* = 33 192 non-obese) in the analyses. The horizontal dotted lines indicate *r* = ±0.02 (corresponds to *r* effects approximately at significance threshold *P* ≤ 4.3e−05). *Abbreviations*: BMI, body mass index; FA, fractional anisotropy; AD, axial diffusivity; RD, radial diffusivity; MD, mean diffusivity; L, left; R, right; *r*, partial correlation coefficient; WC, waist circumference; WHR, waist-to-hip ratio. For regional white matter abbreviations, see Fig. 2.

Multiple linear regression analyses (Step 1) indicated similar associations with DTI metrics across the included obesity phenotypes (Fig. 3; Supplementary Tables 1–4). We generally observed stronger significant effects for the continuous BMI (|*r*| in [0.02, 0.20], *P* in [5.6e−05, 0〉 (i.e. *P*-value range from 5.6e−05 to approximately 0)) than the categorical obesity measure (|*r*| in [0.02, 0.14], *P* in [3.1e−05, 8.1e−185]). The significant effects for WHR (|*r*| in [0.02, 0.13], p in [1.3e−05, 2.1e−142]) and waist circumference (|*r*| in [0.02, 0.17], p in [3.0e−05, 1.5e−246]) were overall lower than for BMI, while the range of effects for obesity fell between that of WHR and waist circumference. The number of significant ROIs was slightly higher for BMI (78.1%) than for obesity (76.0%), and we observed slightly higher numbers of significant ROIs for both WHR (82.3%) and waist circumferences (80.7%), possibly reflecting greater sensitivity to abdominal fat than both BMI and obesity. In general, we observed the highest numbers of significant ROIs for FA (obesity: 87.5%; BMI: 87.5%; WHR: 83.3%; waist circumference: 93.8%; except higher numbers for WHR and AD) and similar numbers for MD (obesity: 75.0%; BMI: 79.2%; WHR: 79.2%; waist circumference: 75.0%) across obesity phenotypes. For RD (obesity: 72.9%; BMI: 75.0%; WHR: 81.3%; waist circumference: 72.9%) and AD (obesity: 68.8%; BMI: 70.8%; WHR: 85.4%; waist circumference: 81.3%), we observed some variations, with greater numbers of significant ROIs for WHR and waist circumference than for obesity and BMI.

We observed an overall pattern of most significant effects with *brainstem tracts* across the included obesity phenotypes, with mixed effect directionality for FA, and predominantly lower AD, RD, and MD effects (Fig. 3). For FA, the most significant associations were with higher pontine and lower superior cerebellar peduncle effects. The corticospinal tract and medial lemniscus also exhibited significantly higher or non-significant FA effects, while the inferior and middle cerebellar peduncle exhibited lower FA effects. Additionally, we further observed the overall lowest AD and RD effects for the pontine followed by the corticospinal tract—the same tracts with the significantly higher FA effects—and the superior cerebellar peduncle (only AD), the tract with the lowest FA effect. MD showed the overall lowest effects for the pontine, followed by the corticospinal tract and medial lemniscus before the superior cerebellar peduncle. Although there were regional variations in the association between the individual obesity phenotypes and brainstem tracts, these findings suggest a general picture of an inverse association between obesity phenotypes and lower directional and overall diffusion for brainstem tracts, as well as lower perpendicular diffusion for the corticospinal tract, pontine, and medial lemniscus—the tracts with significantly higher or non-significant FA effects.

For *projection*, *commissural*, and *association pathways*, the individual obesity phenotypes were associated with predominantly lower FA effects, together with predominately lower AD and higher RD and MD effects (although several ROIs also show opposing effect directions, particularly for AD; Fig. 3). These findings indicate that various tract properties contribute to lower FA (e.g. predominantly lower AD or higher RD, or in combination) for obesity phenotypes. The effect sizes were smaller than for brainstem tracts, and the patterns not as clear, making it more challenging to distinguish generalizable patterns for non-brainstem tracts.

The sensitivity analyses show that the core results remained the same, albeit with fewer significant regions (obesity: 62.0%; BMI: 67.2%; WHR: 79.2%; waist circumference: 70.8%) and slight modification of effect size and *P*-value ranges (obesity: *r* in [0.02, 0.14], *P* in [3.4e−05, 1.64e−174]; BMI: *r* in [0.02, 0.19], *P* in [4.2e−05, 0〉; WHR: *r* in [0.02, 0.12], *P* in [2.1e−05, 1.4e−118]; waist circumference: *r* in [0.02, 0.16], *P* in [3.7e−05, 1.5e−234]), when adjusting for self-reported comorbid cardiometabolic factors (i.e. diagnosis of diabetes, hypertension, and high cholesterol, and current smoking and alcohol consumption; Supplementary Fig. 3; Supplementary Tables 5–8).

### Interaction effects between sex and obesity phenotypes on DTI metrics

**Figure 4:**
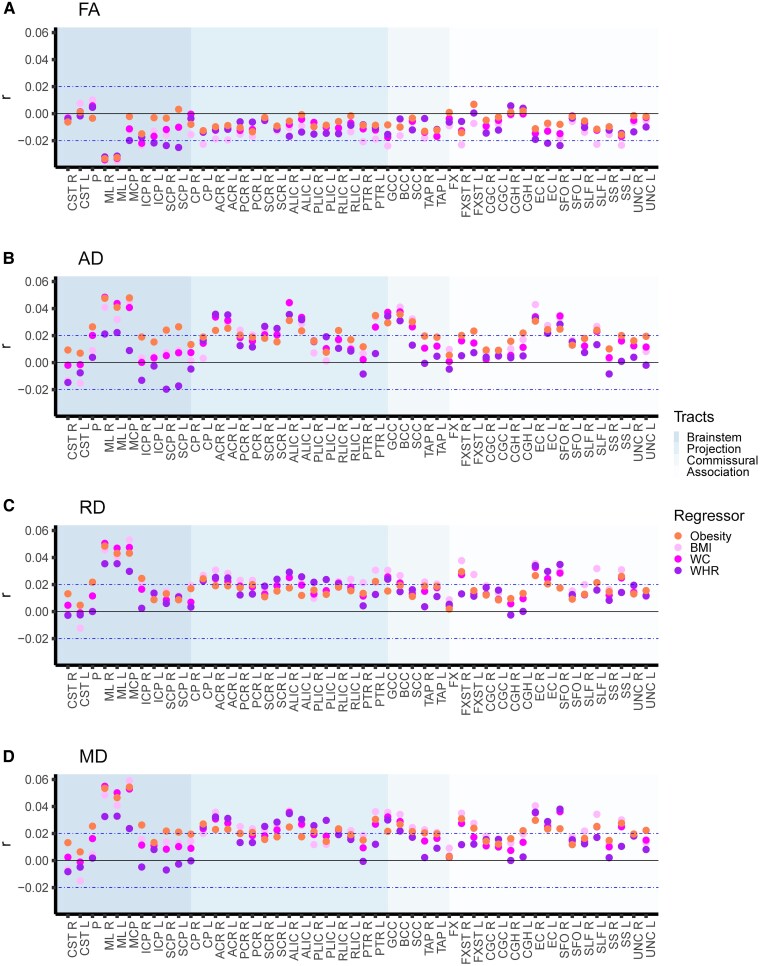
Interaction effects between sex and obesity phenotypes on diffusion tensor imaging metrics. The figure shows the interaction effects between sex (female reference) and (i) obesity versus non-obesity; (ii) BMI; (iii) waist circumference; and (iv) WHR separately on diffusion tensor imaging metrics for (**A**) FA, (**B**) AD, (**C**) RD, and (**D**) MD, after adjusting for the corresponding main effects, age, age,^2^ ethnicity, and site. We included 40 040 (52.2% female) UK Biobank participants (*n* = 6912 with obesity (50.7% female); *n* = 33 192 non-obese (52.5% female)) in the analyses. The horizontal dotted lines indicate *r* = ±0.02 (corresponds to *r* effects approximately at significance threshold *P* ≤ 4.3e−05). *Abbreviations*: BMI, body mass index; FA, fractional anisotropy; AD, axial diffusivity; RD, radial diffusivity; MD, mean diffusivity; L, left; R, right; *r*, partial correlation coefficient; WC, waist circumference; WHR, waist-to-hip ratio. For regional white matter abbreviations, see Fig. 2.

**Figure 5:**
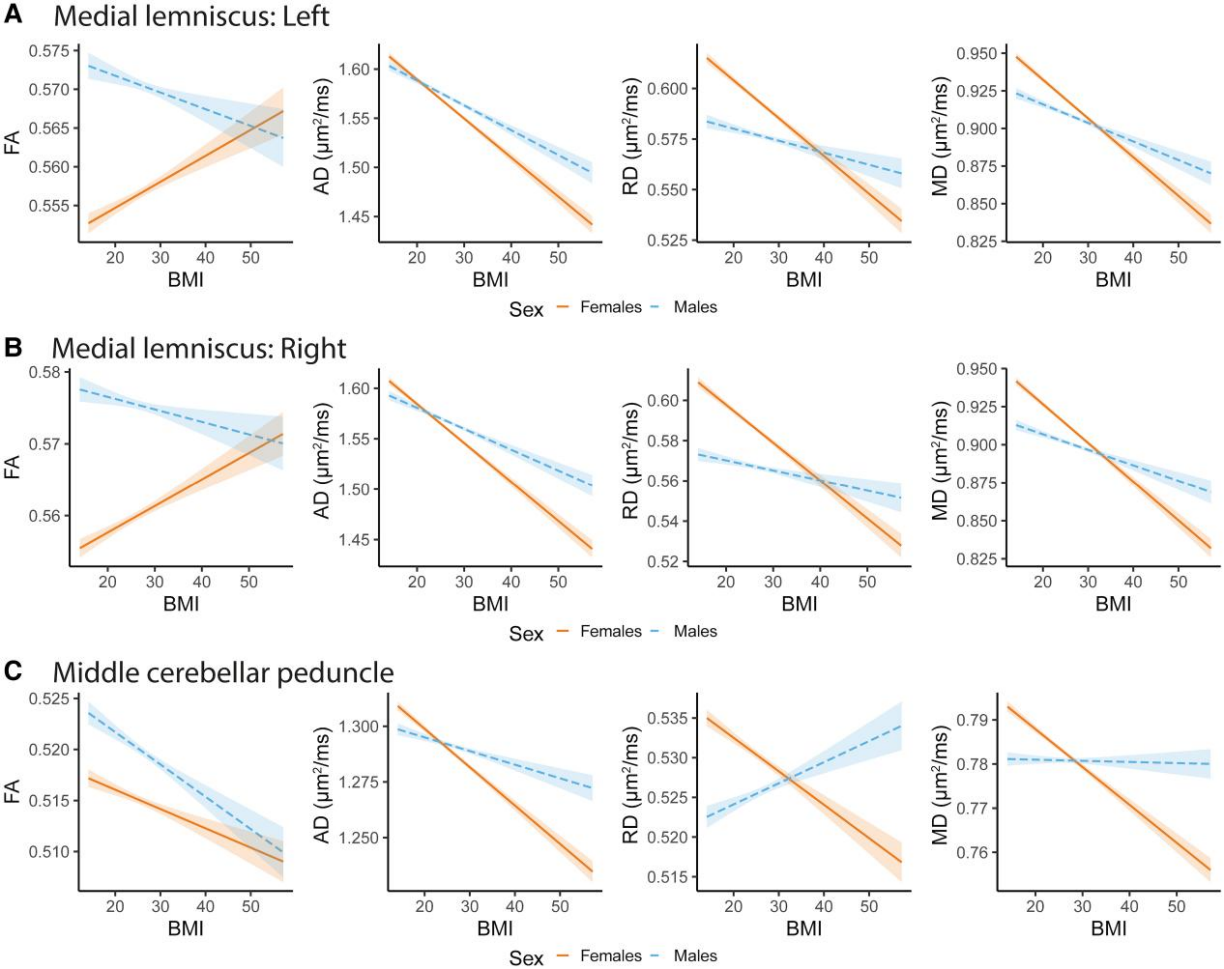
Illustration of interaction effects between sex and BMI for selected regions of interests. Sex-by-BMI interaction effects of the (**A**) left medial lemniscus, (**B**) right medial lemniscus, and (**C**) middle cerebellar peduncle using the interact_plot R-function after adjusting for the corresponding main effects, age, age^2^, ethnicity, and site (BMI uncentered) and 95% confidence intervals. We included 40 040 (52.2% female) UK Biobank participants in the analyses. The figures show the effects/fitted lines with confidence/predicted intervals. AD, RD, and MD are in square micrometer per millisecond (μm2/ms), while FA is a dimension-less metric. *Abbreviations*: BMI, body mass index; FA, fractional anisotropy; AD, axial diffusivity; RD, radial diffusivity; MD, mean diffusivity; L, left; R, right; *r*, partial correlation coefficient.

Sex by obesity phenotypes analyses (Step 2) revealed significant interaction effects for a substantial number of ROIs across DTI metrics (obesity: 32.3%, |*r*| in [0.02, 0.05], *P* in [4.1e−05, 1.4e−27]; BMI: 41.2%, |*r*| in [0.02, 0.06], *P* in [3.2e−05, 2.5e−32]; WHR: 27.1%, |*r*| in [0.02, 0.04], *P* in [2.3e−05, 2.7e−14]; waist circumference: 29.7%, |*r*| in [0.02, 0.06], *P* in [4.2e−05, 3.7e−28]; Fig. 4; Supplementary Tables 9–12). The number of significant sex interaction effects varied across DTI metrics, with higher proportion for AD (obesity: 43.8%; BMI: 37.5%; WHR: 27.1%; waist circumference: 35.4%), RD (obesity: 22.9%; BMI: 52.1%; WHR: 31.3%; waist circumference: 35.4%), and MD (obesity: 58.3%; BMI: 56.3%; WHR: 35.4%; waist circumference: 41.7%) than FA (obesity: 4.2%; BMI: 18.8%; WHR: 14.6%%; waist circumference: 6.3%). These findings indicate regional sex-related variation of obesity phenotypes on white matter microstructure. We observed the overall largest interaction effects for two brainstem tracts, the medial lemniscus (FA, AD, RD, and MD) and the middle cerebellar peduncle (AD, RD, and MD only). For illustration purposes, limiting to the continuous BMI measure, we further show the interaction effects between sex and BMI for these two tracts (Fig. 5A–C). The illustrations show that for the medial lemniscus, the higher FA and lower AD, RD, and MD effects at higher BMI are attenuated in males relative to females. For the middle cerebellar peduncle, we observed a similar pattern for AD and MD, suggesting attenuated reduction at higher BMI in males relative to females, while we observed opposing effects for RD.

In the sensitivity analyses, where we additionally adjust for self-reported cardiometabolic comorbid factors, we observed a similar pattern of significant interaction effects between sex and obesity phenotypes on DTI metrics (obesity: 29.2%, |*r*| in [0.02, 0.05], *P* in [3.3e−05, 3.9e−27]; BMI: 28.1%, |*r*| in [0.02, 0.06], *P* in [3.6e−05, 1.7e−30]; WHR: 23.4%, |*r*| in [0.02, 0.04], *P* in [4.3e−05, 2.9e−12]; waist circumference: 22.4%, |*r*| in [0.02, 0.06], *P* in [4.3e−05, 4.9e−28]; Supplementary Fig. 4; Supplementary Tables 13–S16).

### Interaction effects between age and obesity phenotypes on DTI metrics

**Figure 6:**
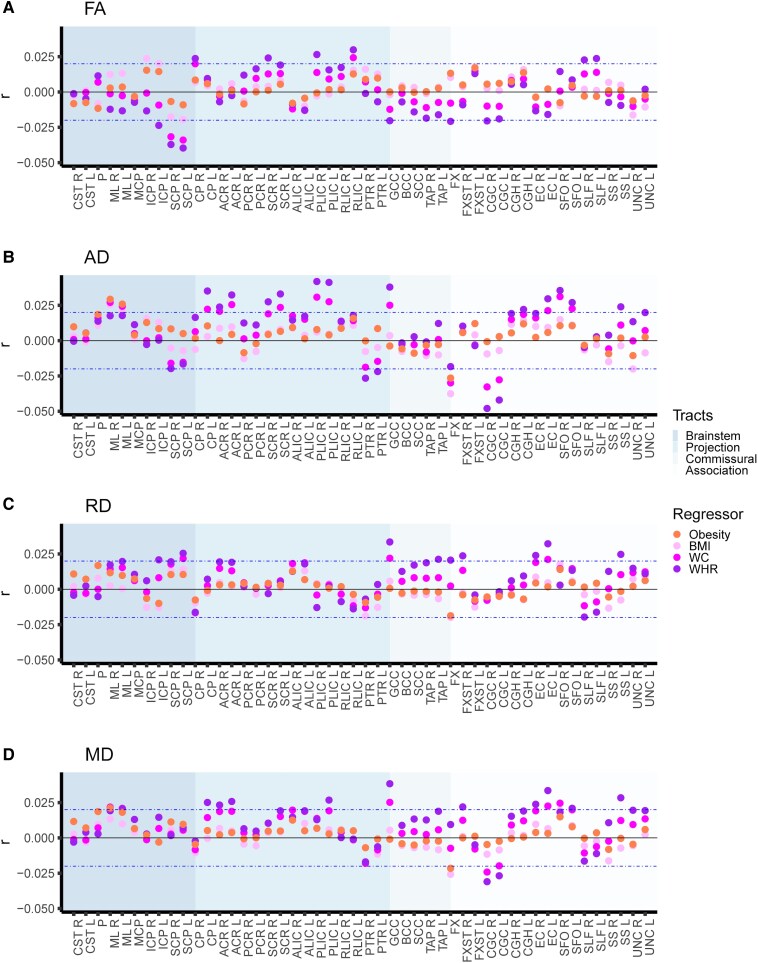
Interaction effects between age and obesity phenotypes on diffusion tensor imaging metrics. The figure shows the interaction effects between age and (i) obesity versus non-obesity; (ii) BMI; (iii) waist circumference; and (iv) WHR separately on diffusion tensor imaging metrics for (**A**) FA, (**B**) AD, (**C**) RD, and (**D**) MD, after adjusting for the corresponding main effects, and sex, ethnicity, and site. We included 40 040 UK Biobank participants (*n* = 6912 with obesity; *n* = 33 192 non-obese) in the analyses. The horizontal dotted lines indicate *r* = ±0.02 (corresponds to *r* effects approximately at significance threshold *P* ≤ 4.3e-05). *Abbreviations*: BMI, body mass index; FA, fractional anisotropy; AD, axial diffusivity; RD, radial diffusivity; MD, mean diffusivity; L, left; R, right; *r*, partial correlation coefficient; WHR, waist-to-hip ratio; WC, waist circumference. For regional white matter abbreviations, see Fig. 2.

Lastly (Step 3), the age by obesity phenotypes analyses revealed significant interactions for some ROIs across DTI metrics (obesity: 2.6%, |*r*| in [0.02, 0.03], *P* in [2.3e−05, 4.5e−09]; BMI: 2.6%, |*r*| in [0.02, 0.04], *P* in [2.4e−05, 5.4e−14]; WHR: 26.0%, |*r*| in [0.02, 0.05], *P* in [4.2e−05, 7.0e−22]; waist circumference: 14.1%, |*r*| in [0.02, 0.03], *P* in [3.3e−05, 1.0e−11]). We observed the highest number of significant ROIs for AD (obesity: 6.3%; BMI: 6.3%; WHR: 35.4%; waist circumference: 31.3%), followed by MD (obesity: 4.2%; BMI: 2.1%; WHR: 27.1%; waist circumference: 12.5%), FA (obesity: 0%; BMI: 2.1%; WHR: 22.9%; waist circumference: 6.3%), and RD (obesity: 0%; BMI: 2.1%; WHR: 18.8%; waist circumference: 6.3%). Thus, there were a greater number of significant ROIs for WHR and waist circumference with stronger and more widespread interaction effects with age particularly for WHR (Fig. 6; Supplementary Tables 17–20) than observed for obesity and BMI, possibly reflecting greater sensitivity to abdominal obesity for both WHR and waist circumference.

**Figure 7:**
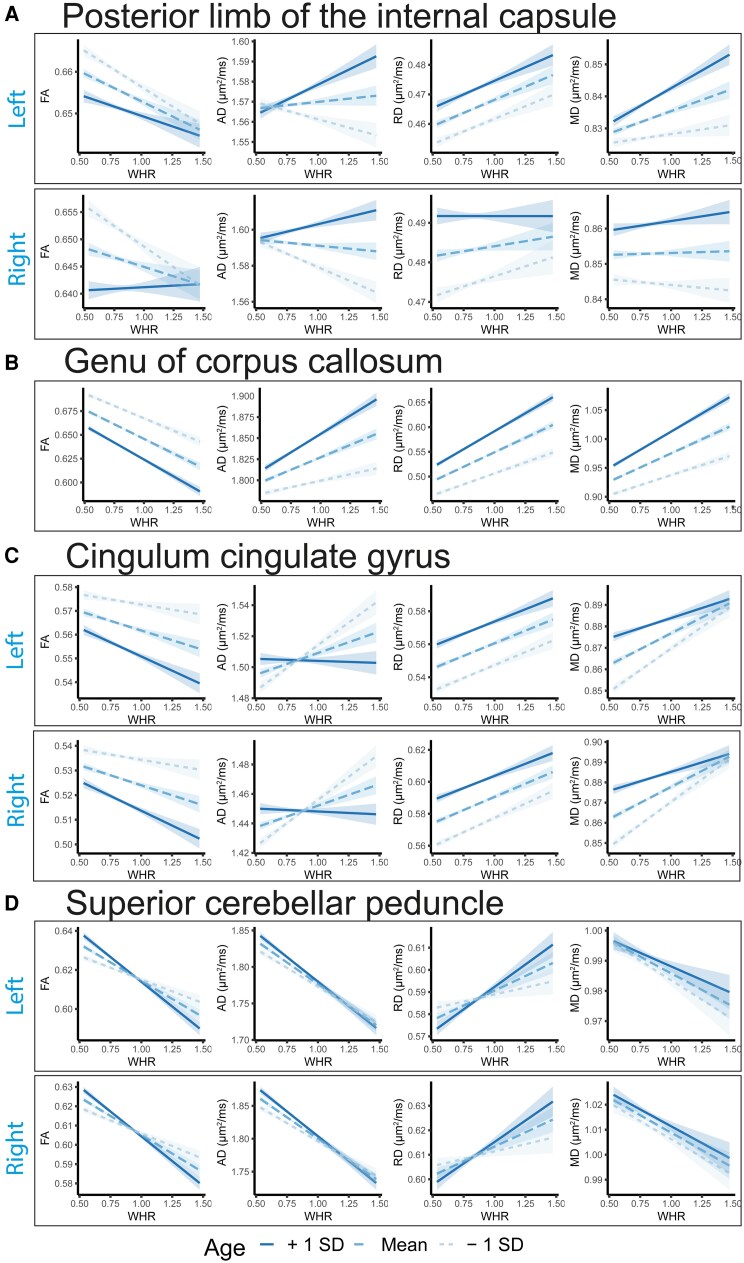
Illustration of WHR by age interaction on white matter regions of interests. A–D shows the interaction effects between WHR and age on selected regions of interests using the interact_plot R-function (WHR and age uncentered, with 95% confidence intervals). The statistical model was adjusted for the main effect of WHR and age, sex, ethnicity, and site. We included 40 040 UK Biobank participants in the analyses. AD, RD, and MD are in square micrometer per millisecond (μm^2^/ms), while FA is a dimension-less metric. *Abbreviations*: FA, fractional anisotropy; AD, axial diffusivity; RD, radial diffusivity; MD, mean diffusivity; WHR, waist-to-hip ratio.

In the following, for illustration purposes, we focused on the tracts with the most significant interactions between WHR and age on DTI metrics (Fig. 6; Supplementary Table 20). We observed the overall highest interaction effects between WHR and age for AD of the *posterior limb of the internal capsule* and AD and MD of the *genu of corpus callosum*, and the overall lowest interaction effect sizes for the AD of the *cingulum cingulate gyrus* and FA of the *superior cerebellar peduncle*. The interpretation of the results varies for the various tracts—all depending on the main effect of WHR. For the *posterior limb of the internal capsule*, there is a significant interaction effect between age and WHR despite no main effect of WHR (Fig. 7A). Contrastingly, for the *genu of the corpus callosum*, the main effect of WHR is substantial, and the significant interaction with age suggests steeper age-related alterations at higher WHR at higher ages (across FA, AD, RD, and MD) (Fig. 7B). For the *cingulum*, the main effect of WHR was attenuated by the negative interaction with age and became weaker at higher ages (Fig. 7C). Like the genu of the corpus callosum, the negative main effect of the *superior cerebellar peduncle* (FA and AD) was substantial, and the results further indicate steeper alteration at higher WHR and higher ages (Fig. 7D).

The sensitivity analyses, where we additionally adjusted for self-reported cardiometabolic comorbid factors, show similar results with only minor adjustments of the effect sizes and significance levels on DTI metrics (obesity: 2.6%, *r* in [0.02, 0.03], *P* in [2.8e−05, 3.7e−09]; BMI: 4.2%, *r* in [0.02, 0.04], *P* in [1.8e−05, 6.7e−15]; WHR: 25.0%, *r* in [0.02, 0.05], *P* in [4.2e−05, 5.2e−22]; waist circumference: 13.5%, *r* in [0.02, 0.03], *P* in [4.1e−05, 2.7e−11]; Supplementary Fig. 5; Supplementary Tables 21–24).

## Discussion

With this study we aimed to elucidate whether and how obesity phenotypes relate to regional brain white matter phenotypes, as well as putative sex- and age-related differences. We illustrated that there are (i) widespread obesity-related associations with regional brain white matter phenotypes across DTI metrics, with (ii) the largest effects for brainstem tracts; and that there are (iii) sex- and age-interactions with obesity phenotypes on white matter phenotypes, with (iv) more widespread sex- than age-related interaction effects. Lastly, (v) the sensitivity analysis verifies that these results are robust even when adjusting for additional self-reported cardiometabolic comorbidities. These findings support a negative link between higher measures of obesity phenotypes and widespread regional white matter microstructure alterations with largest effects for the brainstem that may differ based on sex and age. In turn, these findings may relate to the observed comorbid link between cardiometabolic conditions and brain disorders, as well as sex- and age-related variations in brain disorders, and may imply interactive metabolic processes and body fat-related pathways between physical and brain health.

Our most striking finding is the relatively strong associations between obesity phenotypes and brainstem tracts of generally lower AD, RD, and MD, which may culminate in either lower or higher FA at higher measured obesity phenotypes, all depending on the underlying diffusion properties. The revealed patterns are in line with a prior study using a partly overlapping sample,^4^ and further suggests axonal degeneration with little indications of demyelination and inflammatory processes (as indicated by lower AD, RD, and MD)^16^ in the brainstem in obesity, irrespective of whether FA is higher or lower. Brainstem tracts connect the spinal cord, brainstem, cerebellum, subcortical structures (e.g. thalamus), and/or cerebrum.^48^ Although speculative, the observed patterns may implicate the brainstem body–brain communication hub in obesity. Indeed, they may relate to impairments in the brainstem’s role in regulating appetite and food intake in obesity, given that nutritional information is passed from the gut via vagal sensory nerves to the brainstem and hormones produced in the gut are linked to nutrient absorption and metabolism and appetite regulation in the brain.^49^ Another possible explanation is the involvement of the sensorimotor system, where a more sedentary lifestyle in obesity may result in less maintenance or development, or more degeneration, of the important brainstem body–brain communication pathways. Indeed, we have previously shown brainstem and cerebellum volumes alterations for sarcopenic traits^18^ and obesity phenotypes^50^ in partly overlapping samples. Furthermore, structures of the sensorimotor system—including the brainstem and cerebellum—mediated the link between sarcopenic traits and lower cognitive performance,^18^ and similar patterns may explain the observed link between midlife obesity and later cognitive deficits.^51^ Notably, however, these structures are often not included in large-scale studies of brain disorders, with some exceptions (e.g. corticospinal tract or in studies of ataxia).^10^ Given the overrepresentation of cardiometabolic comorbidities in brain disorders^1-3^—suggestive of a link between physical and brain health—it is paradoxical that structures connecting the body with the brain, and vice versa, are often not included in larger studies. Thus, future large-scale studies of brain disorders that target structures interconnecting the body with the brain, and vice versa, are warranted to enhance our understanding of brain disorders and the physical, cardiometabolic, link.

We also found that obesity phenotypes are associated with widespread regional white matter phenotypes across DTI metrics. Indeed, the findings suggests that the obesity connection with white matter is more systemic than localized, with widespread associations, albeit with the strongest effects for brainstem tracts. The direction of effects varied by region, but for non-brainstem tracts the predominant picture is of lower FA in combination with predominantly lower AD and higher RD and MD (albeit with some mixed effects for AD and MD), which may indicate a pattern of regional axonal degeneration, demyelination, and inflammatory processes (as indicated by lower AD, and higher RD and MD)^16^ of non-brainstem tracts in obesity. These findings align with prior studies showing lower FA at higher BMI or WHR^4-8^ and with observations from smaller studies suggesting axonal degeneration and demyelination for obesity phenotypes.^5^ However, to our knowledge, no prior large-scale study has comprehensively shown indications of brain axonal degeneration, demyelination, and inflammatory processes in obesity for non-brainstem tracts.

Furthermore, our results show that sex is an essential factor that needs consideration when addressing obesity phenotypes and white matter microstructure, which may relate to sex differences in healthy body fat percentage and body fat distributions, as well as hormonal status. The range of normal body fat is higher for females (15–30%) than for males (10–20%).^28^ Females also naturally accumulate more body fat across the hips and thighs, while males often accumulate body fat across the trunk and abdomen,^11^ which in turn may be influenced by hormonal status.^52^ Indeed, males generally have higher levels of visceral fat than females, although menopausal and post-menopausal females may shift to accumulate body fat more similarly to males.^11^ This may suggest different body fat-related ageing trajectories in males and females, with the additional complexity of menopause for females. Thus, understanding the observed sex differences in how obesity phenotypes relate to white matter microstructure might be important for disentangling sex-specific physical and brain disease risks and outcomes.

We also found regional age-by-obesity phenotypes interactions on white matter microstructure, with larger effects for measures sensitive to abdominal obesity. This may relate to age-related increases in abdominal obesity particularly for males and postmenopausal females,^11^ suggestive of sex-specific ageing trajectories that might also be reflected in brain structure and possibly in disorders. Such interaction effects between indicators of abdominal obesity and age on white matter microstructure may form the foundation for future works, that also targets putative sex-specific ageing trajectories, the added complexity of menopause in females, and links to health outcomes in ageing.

Taken together, our findings support that cardiometabolic factors are of importance for brain health. However, we do not know the causal direction of effects, and indications of reciprocal complex mechanisms exist. Cardiometabolic factors, heart phenotypes, and cardiometabolic multimorbidity are associated with white matter phenotypes, cognitive functioning, and several brain disorders.^1,2,53-56^ Similarly, several brain disorders are associated with cardiometabolic factors, cognitive functioning, and white matter phenotypes.^1,2,10,55,57,58^ Danish population registry studies further suggest that most categories of physical conditions show higher risk for developing mental disorders,^59^ and likewise that most mental disorders are associated with higher risk for developing physical conditions.^60^ Additionally, we have illustrated that brain white matter phenotypes mediates the link between sarcopenic-traits^18^ or accumulated liver fat^53^ and lower cognitive performance, and a recent review suggested elevated prevalence of physical conditions in major depression disorder, and vice versa.^2^ These observations suggest interconnected and reciprocal links between the body and the brain that may involve common body fat-related pathways, and may indicate that a whole-body approach and novel treatment strategies capturing the body–brain link (e.g. possibly GLP1-RA^61^ or similar drugs) may be beneficial for treating comorbid brain and cardiometabolic and other physical conditions.

This study offers notable strengths. We included a well-characterized sample of unprecedented size to robustly capture small effects^47^; this is consistent with prior related research using partly overlapping samples,^18,50,53^ earlier works showing that large samples are needed to detect small effects,^50,62^ and that such small effects are common in brain imaging research.^50,62,63^ We performed analyses across commonly used conventional DTI metrics, modelled sex and age specific effects, and illustrated that several DTI metrics are needed to disentangle the underlying tissue properties. Furthermore, the sensitivity analyses show that the results are robust even when adjusting for self-reported cardiometabolic comorbid factors. The study addresses gaps in the scientific literature and provides novel insight into the complexity linking sex, age, and obesity phenotypes with white matter microstructure, which may be relevant for health and disease trajectories. The study also had some limitations. The sample is healthier than the general population^64^ and predominantly includes self-identified white Europeans. It was beyond the study’s scope to investigate body composition measures, other cardiometabolic risk factors, specific clinical comorbid conditions, metabolically healthy and unhealthy obesity,^28^ oxidative stress and inflammatory processes,^65^ and putative shared genetic factors^2,56^ with white matter phenotypes; all factors that may provide added and more specific information. We implemented a cross-sectional design, although the UKB has data available for investigating baseline or longitudinal changes in obesity phenotypes with DTI phenotypes at the follow-up imaging timepoint. We focused our analyses on the conventional DTI model,^15^ but other dMRI analysis models (e.g. restriction spectrum imaging,^66^ neurite orientation dispersion and density imaging^67^) may provide complementary information.^68^ We did not investigate whether white matter lesions influence the results although our previous work show that cardiometabolic factors (including BMI) are associated with later occurrence of white matter hyperintensities in a partly overlapping sample.^53^ We used multiple linear regression for the statistical analyses, but future work may consider using multimodal techniques that reduce the dimensionality of the data and facilitate interpretation.^69^ We focused on the most significant findings and overall patterns, but the vast number of significant findings suggests that future studies are needed to disentangle the regional complex interplay of sex, age, and obesity phenotypes—as well as the role of individual body composition measures—on white matter microstructure, and putative links to disease onset, progression, and outcome.

To conclude, we observe that obesity phenotypes are associated with widespread white matter microstructure phenotypes, with the largest effects for brainstem tracts; a key hub interconnecting the brain with the rest of the body (and vice versa) that is involved in appetite regulation. Furthermore, our analysis indicated more widespread sex- than age-related interaction effects with obesity phenotypes on white matter phenotypes in this sample. Our findings support a link between cardiometabolic and brain health and suggest that addressing obesity-related risk factors may be important for prevention, treatment, and outcome of comorbid physical and brain disorders, although further studies are needed to examine causal effects.

## Supplementary Material

fcag026_Supplementary_Data

## Data Availability

The UK Biobank resource is open for eligible researchers upon application (http://www.ukbiobank.ac.uk/register-apply/). We used publicly available resources to process the brain image data and conduct statistical analyses. We extracted data from individual UK biobank baskets using the *ukb_helper.py* script (https://github.com/precimed/ukb). The project R-scripts are publicly available at OSF.io (https://doi.org/10.17605/OSF.IO/CUS4X).
