# Supplementary material for: Linking obesity with white matter microstructure highlights the importance of brainstem tracts and sex differences
Source: Brain Commun. 2026 Jan 30;8(1):fcag026. doi: 10.1093/braincomms/fcag026 (PMC12914739; doi:10.1093/braincomms/fcag026)
Supplement: fcag026_Supplementary_Data [file fcag026_supplementary_data.zip › Supplementary_Materials.pdf]

SUPPLEMENTARY MATERIALS

LINKING OBESITY WITH WHITE MATTER MICROSTRUCTURE HIGHLIGHTS THE IMPORTANCE OF BRAINSTEM TRACTS AND SEX DIFFERENCES

TABLE OF CONTENTS

Supplemental Figures **........ 2**

Supplementary Figure 1: Flowchart - Overview of the participant inclusion pipeline. 2

Supplementary Figure 2: Density plots of the included continuous variables: imaging assessment. 3

Supplementary Figure 3: Obesity phenotypes with diffusion tensor imaging metrics – sensitivity analyses adjusting for cardiometabolic comorbid factors. 4

Supplementary Figure 4: Interaction effects between sex and obesity phenotypes on diffusion tensor imaging metrics – sensitivity analyses adjusting for cardiometabolic comorbid factors. 5

Supplementary Figure 5: Interaction effects between age and obesity phenotypes on diffusion tensor imaging metrics – sensitivity analyses adjusting for cardiometabolic comorbid factors. 6

Supplemental Note **........ 7**

Supplementary Note 1: Overview of extracted UK Biobank field IDs. 7

SUPPLEMENTAL FIGURES

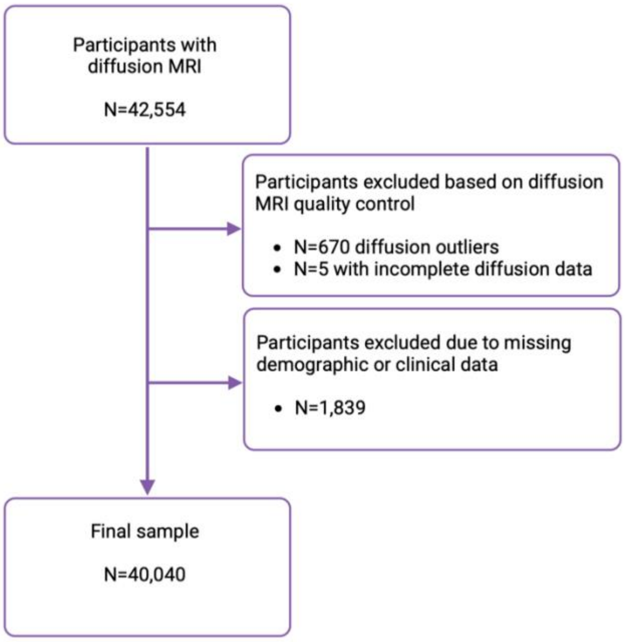

Supplementary Figure 1: Flowchart - Overview of the participant inclusion pipeline. Notes: Created with BioRender. Gurholt, T. (2026) https://BioRender.com/u90f555

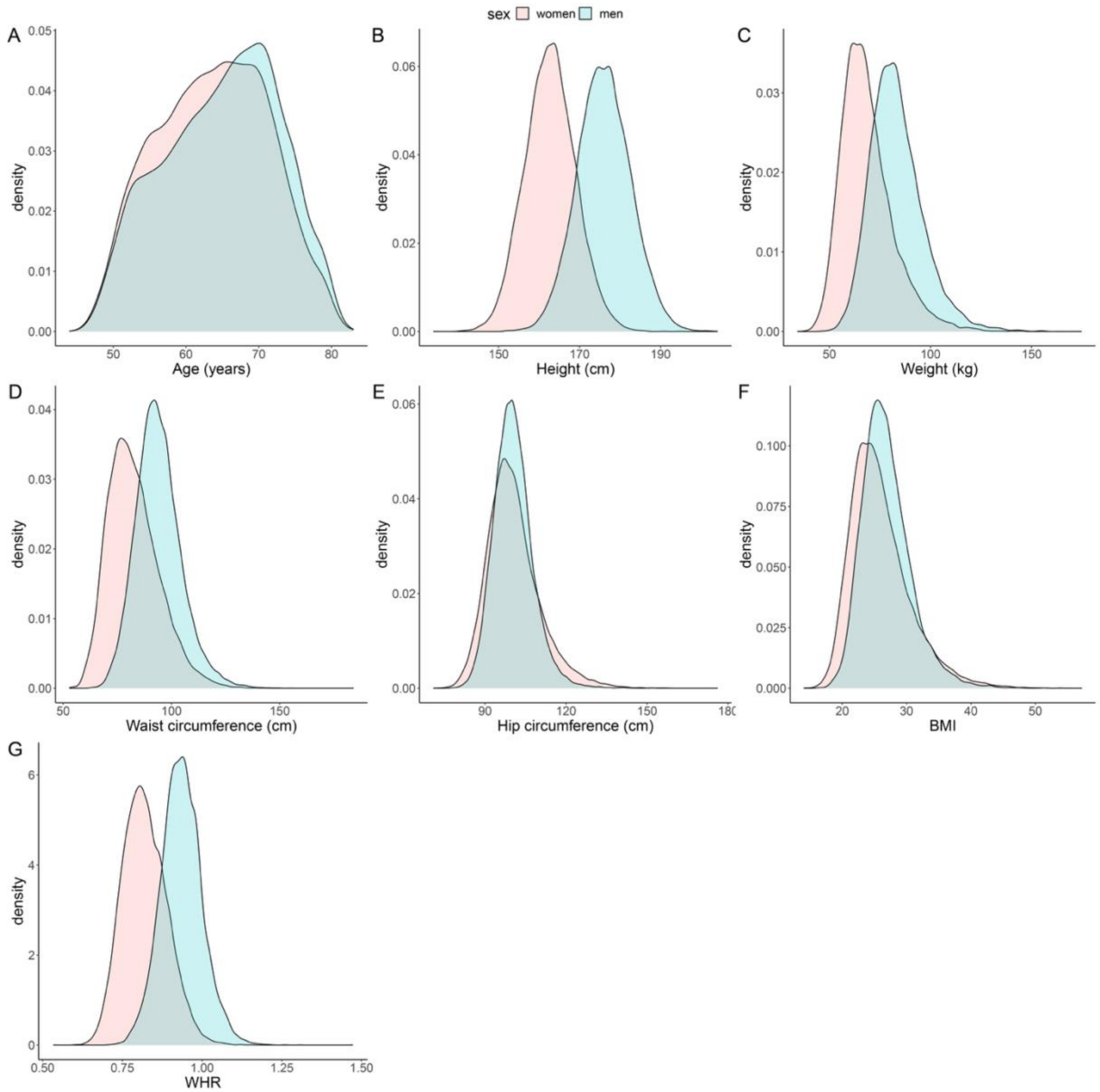

Supplementary Figure 2: Density plots of the included continuous variables: imaging assessment. Notes: The figure shows the density plots split on sex from the imaging timepoint for A) age, B) height, C) weight, D) waist circumference, E) hip circumference, F) BMI, and G) WHR. The density plots correspond to *kernel density estimates*, which is a smoothed version of the histogram, with an area under the curve of 1. The density plots were obtained with R-functions *ggplot* and *geom_density*, using default kernel settings, and were derived based on data from 40,040 UK Biobank participants. Abbreviations: BMI – body mass index; WHR – waist-to-hip ratio.

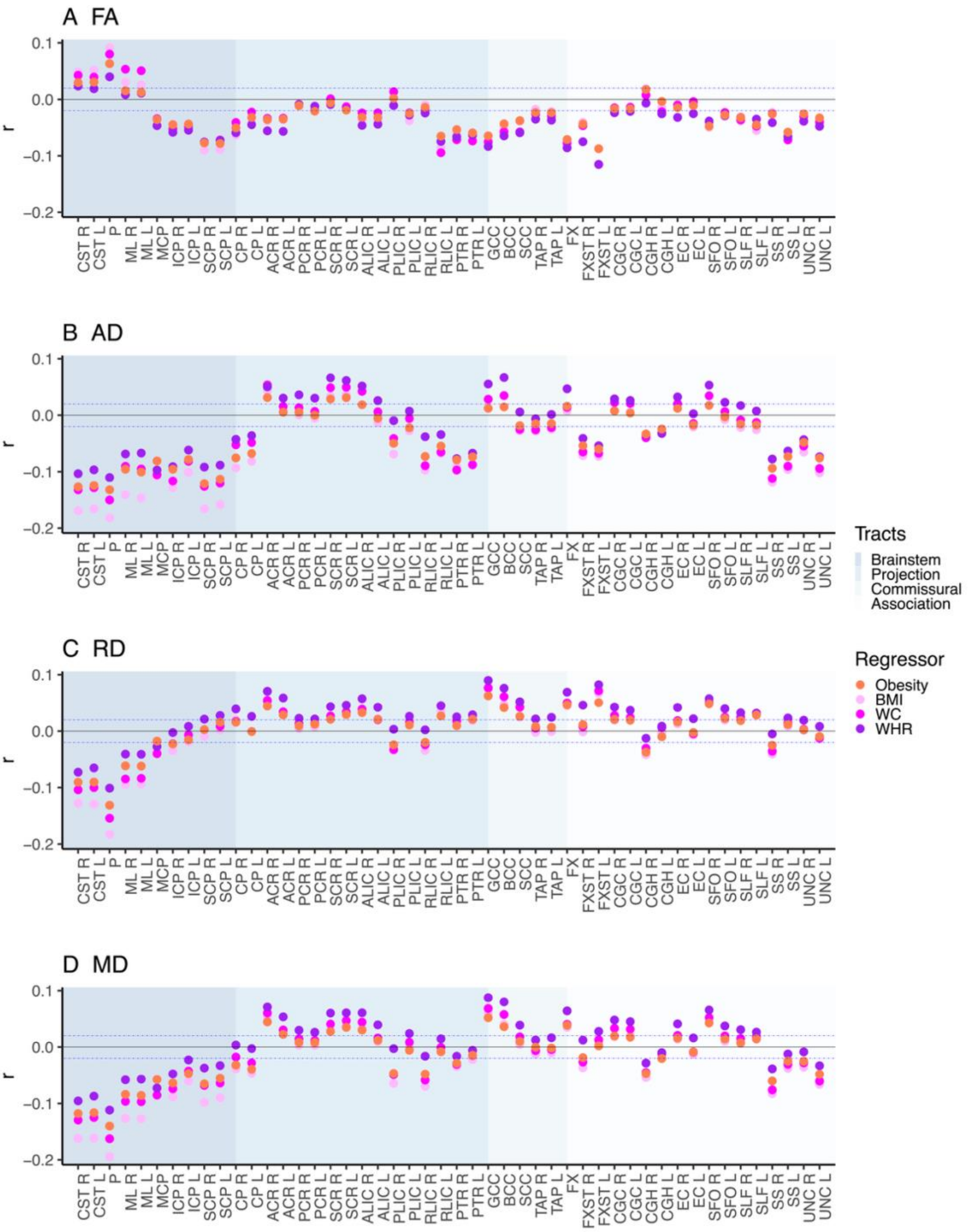

Supplementary Figure 3: Obesity phenotypes with diffusion tensor imaging metrics – sensitivity analyses adjusting for cardiometabolic comorbid factors. Notes: The figure shows the multiple linear regression results of (i) obesity vs non-obesity, (ii) BMI, (iii) waist circumference, and (iv) WHR separately on diffusion tensor imaging metrics for A) FA, B) AD, C) RD, and D) MD, after adjusting for age, age2, sex, age-by-sex, age2-by-sex, ethnicity, self-reported cardiometabolic comorbid factors (i.e., diabetes, hypertension, high cholesterol, current smoking, current alcohol consumption), and site. We included 40,040 UK Biobank participants (N=6,912 with obesity; N=33,192 non-obese) in the analyses. The blue dotted lines indicate r=±0.02 (corresponds to r effects approximately at significance threshold p≤4.3e-05). Abbreviations: BMI – body mass index; WC – waist circumference; WHR – waist-to-hip ratio; FA – fractional anisotropy; AD – axial diffusivity; RD – radial diffusivity; MD – mean diffusivity; L – left; R – right; r - partial correlation coefficient; WC – waist circumference; WHR – waist-to-hip ratio. For regional white matter abbreviations, see Fig. 2.

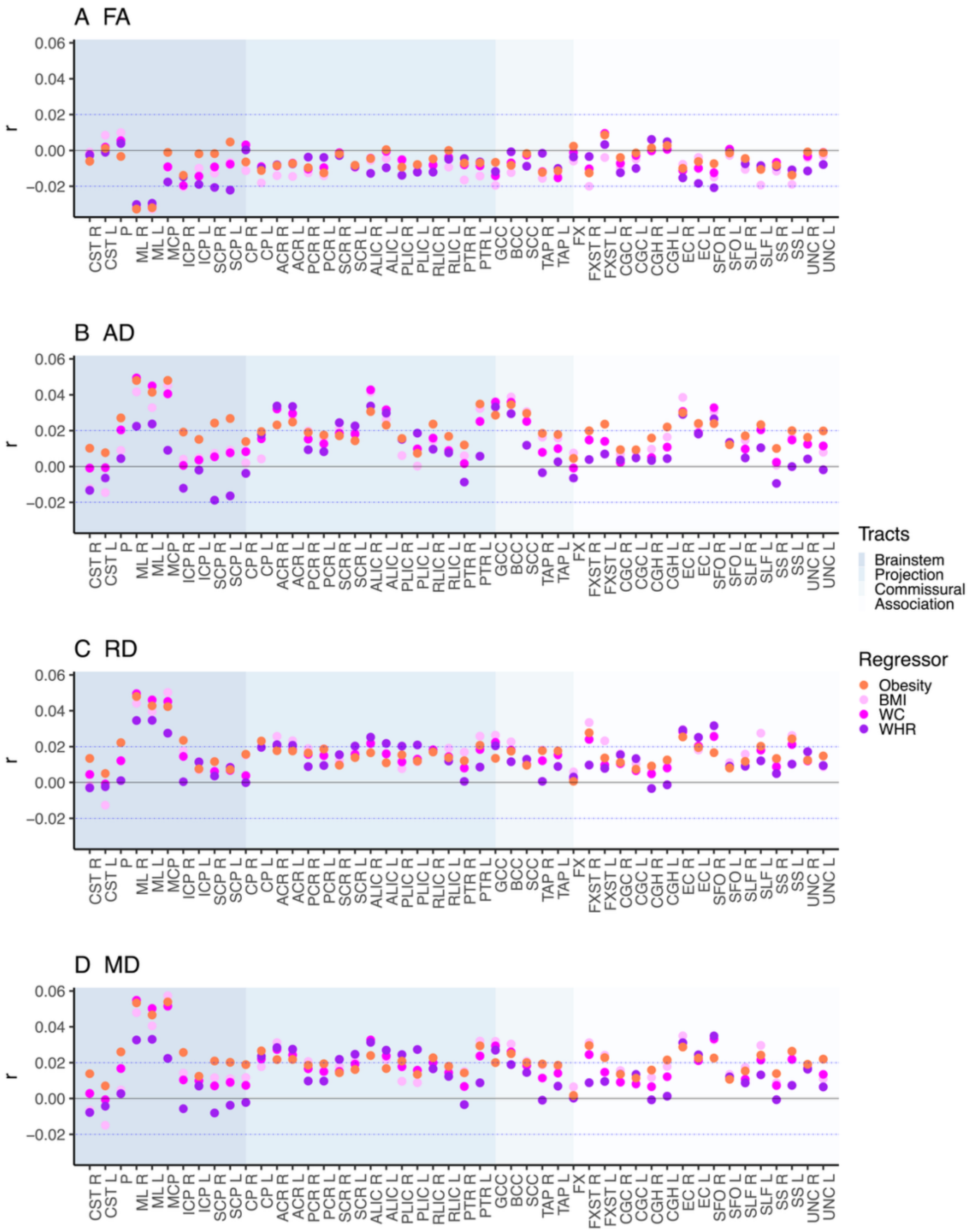

Supplementary Figure 4: Interaction effects between sex and obesity phenotypes on diffusion tensor imaging metrics – sensitivity analyses adjusting for cardiometabolic comorbid factors. Notes: The figure shows the interaction term of the model investigating sex age by (i) obesity vs non-obesity, (ii) BMI, (iii) waist circumference, and (iv) WHR interactions separately on diffusion tensor imaging metrics for A) FA, B) AD, C) RD, and D) MD. We adjusted for the corresponding main effects, age, age^2, ethnicity, self-reported cardiometabolic comorbid factors (i.e., diabetes, hypertension, high cholesterol, current smoking, current alcohol consumption), and site. We included 40,040 UK Biobank participants (N=6,912 with obesity; N=33,192 non-obese) in the analyses. The blue dotted lines indicate r=±0.02 (corresponds to r effects approximately at significance threshold p≤4.3e-05). Abbreviations: BMI – body mass index; WC – waist circumference; WHR – waist-to-hip ratio; FA - fractional anisotropy; AD – axial diffusivity; RD – radial diffusivity; MD – mean diffusivity; L – left; R – right; WC – waist circumference; WHR – waist-to-hip ratio. For regional white matter abbreviations, see Fig. 2.

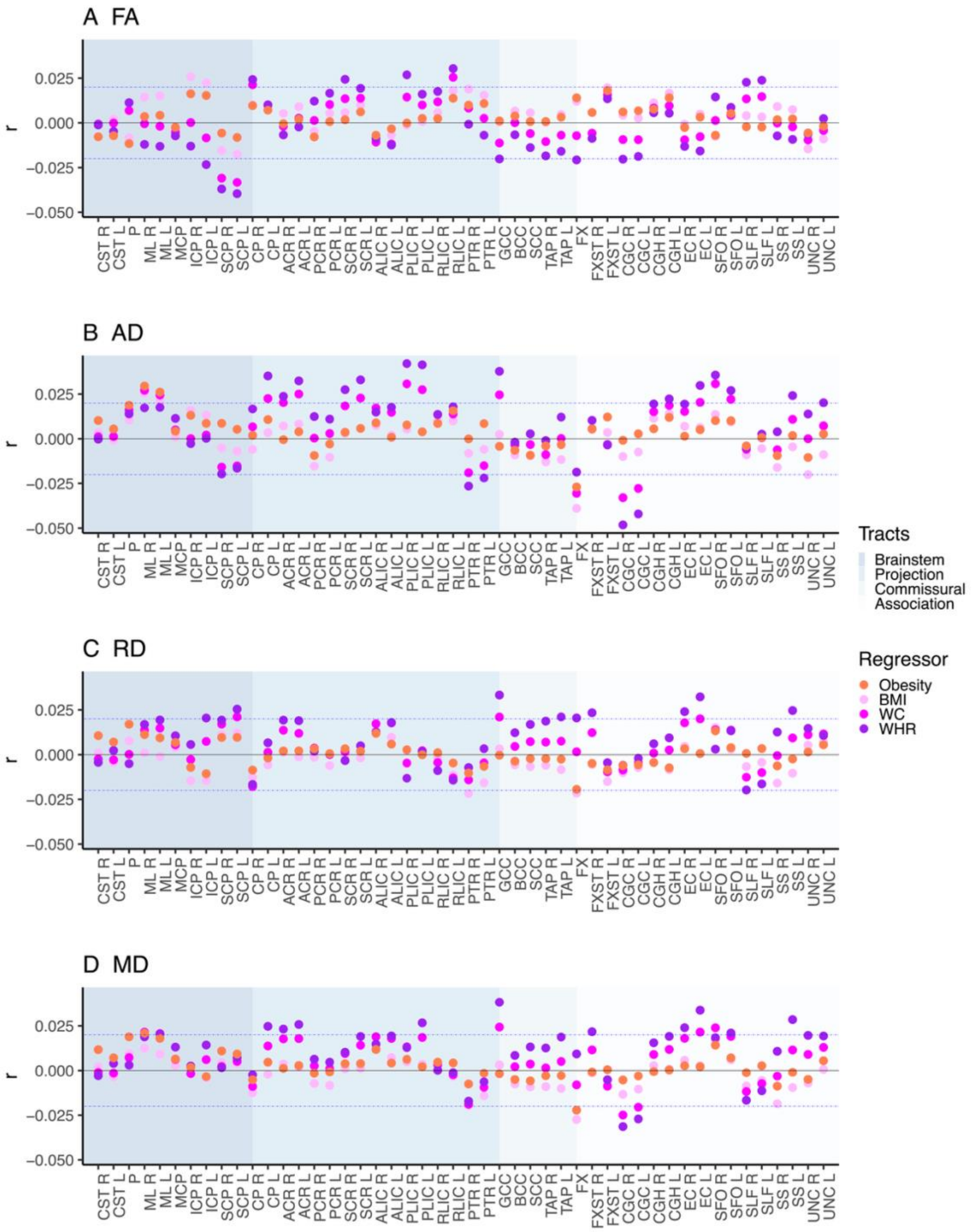

Supplementary Figure 5: Interaction effects between age and obesity phenotypes on diffusion tensor imaging metrics – sensitivity analyses adjusting for cardiometabolic comorbid factors. Notes: The figure shows the interaction term of the model investigating age by (i) obesity vs non-obesity, (ii) BMI, (iii) waist circumference, and (iv) WHR interactions separately on diffusion tensor imaging metrics for A) FA, B) AD, C) RD, and D) MD. We adjusted for the main effects, and age, sex, ethnicity, self-reported cardiometabolic comorbid factors (i.e., diabetes, hypertension, high cholesterol, current smoking, current alcohol consumption), and site. We included 40,040 UK Biobank participants (N=6,912 with obesity; N=33,192 non-obese) in the analyses. The blue dotted lines indicate r=±0.02 (corresponds to r effects approximately at significance threshold p≤4.3e-05). Abbreviations: BMI – Body mass index; FA - fractional anisotropy; AD – axial diffusivity; RD – radial diffusivity; MD – mean diffusivity; L – left; R – right; WC – waist circumference; WHR – waist-to-hip ratio. For regional white matter abbreviations, see Fig. 2.

Supplemental Note

Supplementary Note 1: Overview of extracted UK Biobank field IDs.

Field-ID	Description	Field-ID	Description
31	Sex (from central registry, but participants may update it)	20002	Self-reported non-cancer diagnosis
54	Assessment centre	50	Standing height (cm)
21003	Age (years)	21002	Weight (kg)
20117	Alcohol drinker status (current, previous, never)	21001	BMI (kg/m^2)
20116	Smoking status (current, previous, never)	48	Waist circumference (cm)
21000	Self-reported ethnic background	49	Hip circumference (cm)

Notes: We extracted variables from the imaging time-point when available. For self-reported ethnic background, we complemented missing data with data from the baseline assessment (for details see https://biobank.ndph.ox.ac.uk/showcase/field.cgi?id=21000). Abbreviations: BMI – Body Mass Index.
